# Supplementary material for: Full-Length Envelope Analyzer (FLEA): A tool for longitudinal analysis of viral amplicons
Source: PLoS Comput Biol. 2018 Dec 13;14(12):e1006498. doi: 10.1371/journal.pcbi.1006498 (PMC6314628; doi:10.1371/journal.pcbi.1006498)
Supplement: S1 Text — (PDF) [file pcbi.1006498.s001.pdf]

Supporting Information

Simulation method

The simulation procedure begins with a population of copynumber-weighted HQCS sequences from a real `FLEA` run. The population is augmented with mutants of the input sequences, to ensure that the simulated ground truth population contains sequences that differ by only a few bases. Each HQCS has a $p = 0.2$ probability to donate 30% of its abundance to a closely-related mutant, which contains one, two, or three substitutions with equal probability. This mutated population is treated as the ground truth for all experiments.

In order to simulate sequencing at different depths, different numbers of N reads are drawn from the same ground truth population for each time point. For each value of N (300, 1,000, 3,000, and 10,000 in this paper), and for each time point, N sequences are sampled with replacement from the copynumber-weighted population. Each read is then mutated with an error model derived from true Pacific Biosciences sequences, in order to mimic the errors introduced by sequencing, especially homopolymer length errors.

To simulate a read r from template t, it is necessary to model both r itself and its Phred scores. First an error rate p is drawn from $p \sim Gamma(\alpha = 2, \theta = 0.0017)$. The length n for each run of identical bases in t (including singletons) is lengthened or shortened with equal probability to be $m = max(n \pm \epsilon, 0)$, where $\epsilon \sim Poisson(\lambda = p/c \cdot n^{1.5})$. c is calibration parameter chosen in these experiments to be 1.55 to match observed errors. This process introduces homopolymer length errors, which account for most of the error in Pacific Sciences reads. Then point mutations are introduced at each position with probability $p/4$ of occurring and equal probability for each nucleotide.

Finally, error probabilities are computed for each base as $P = p/4 + m^{1.5}/m$, which is the per-base mutation rate plus a homopolymer error rate. The final simulated Phred scores are obtained by adding error per-base errors $\epsilon \sim \mathcal{N}(0, 0.1)$ in the natural log domain to these probabilities, then converting to Phred scores.

Sequence order for clustering

`USEARCH`'s `cluster_fast` algorithm runs in a single pass, and therefore is sensitive to the order of the input sequences. We investigated four different strategies: none (no re-ordering), shuffle (randomly shuffle the sequences), sort (sort from high to low quality, as measured by expected number of errors), and reverse sort (sort from low to high quality). Ten trials of simulated sequencing were run to generate 3,000 reads. `FLEA` was run on each dataset with all four ordering strategies.

The results clearly favor reverse sorting, as shown in Table A, which does better on average across the ten trials, and in the worst case it does much better. In the worst case, other methods suffer from false negatives, as shown in Table C. We hypothesize that this behavior is caused by reads from the rare templates – which have a low chance of having a high-quality representative read – loading onto the nearest high-quality template.

Table A. EMD score statistics for different ordering strategies, summarized over ten trials.

strategy	min	median	max
none	1.161733	1.754568	4.650523
shuffle	1.042900	1.877201	15.177280
sort	1.362890	2.170702	15.585899
reverse sort	1.077585	1.495208	2.853009

Table B. EMD_{FP} score statistics for different ordering strategies, summarized over ten trials.

strategy	min	median	max
none	0.0	0.012702	0.726121
shuffle	0.0	0.005160	0.634556
sort	0.0	0.019261	0.839726
reverse sort	0.0	0.005992	0.079191

Table C. EMD_{FN} score statistics for different ordering strategies, summarized over ten trials.

strategy	min	median	max
none	0.109267	0.363806	4.064444
shuffle	0.104379	0.359525	13.443283
sort	0.170185	0.623503	13.672487
reverse sort	0.096213	0.233316	1.011137

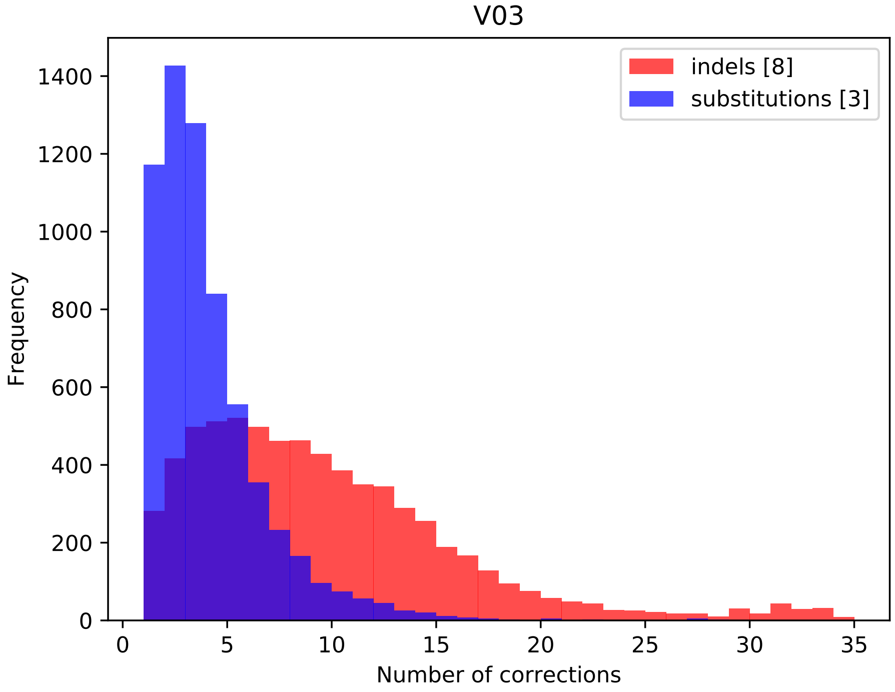

Figure A. Distribution of corrections in V03.

Analysis of error correction in P018 run

To quantify the kinds of corrections FLEA makes, we took each quality controlled CCS sequence and aligned it to its corresponding HQCS. Counting differences reveals far more indels vs substitutions, as expected under the PacBio error model: Fig. A through Fig. F. Different time points, however, have different correction profiles, and it is not clear whether this is due to the behavior of the error correction itself, or varying noise profiles caused by differences during amplification and sequencing.

Additionally, the number of corrections (both substitutions and indels) per CCS sequence correlates extremely strongly with the expected number of errors per sequence, as derived from the QV scores. The Spearman correlation coefficients range between 0.69 and 0.76. Scatter plots are depicted in Fig. G through Fig. L. The number of expected errors becomes discrete for values ≥ 10 in these plots because of rounding in `USEARCH`.

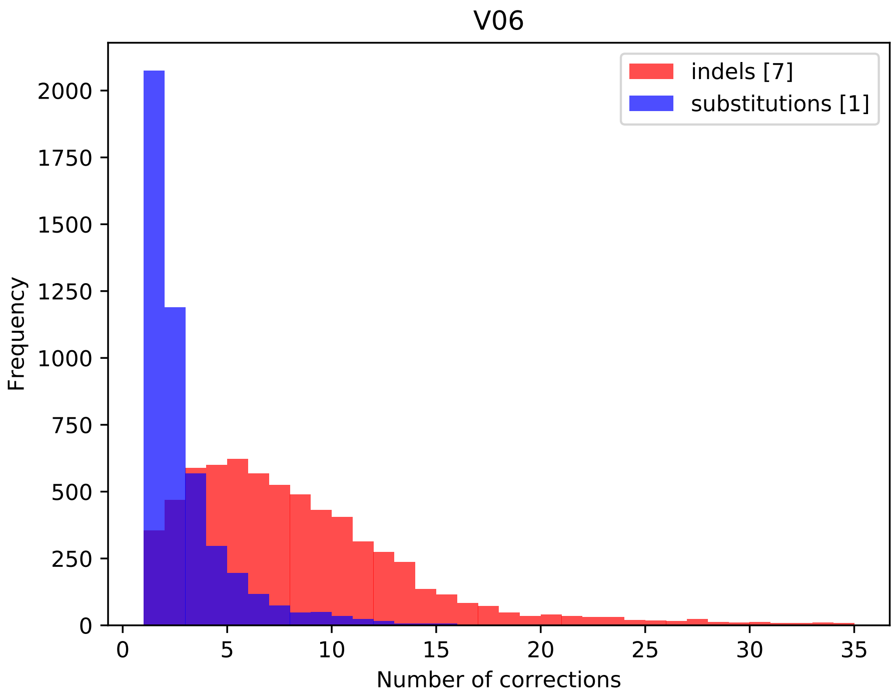

Figure B. Distribution of corrections in V06.

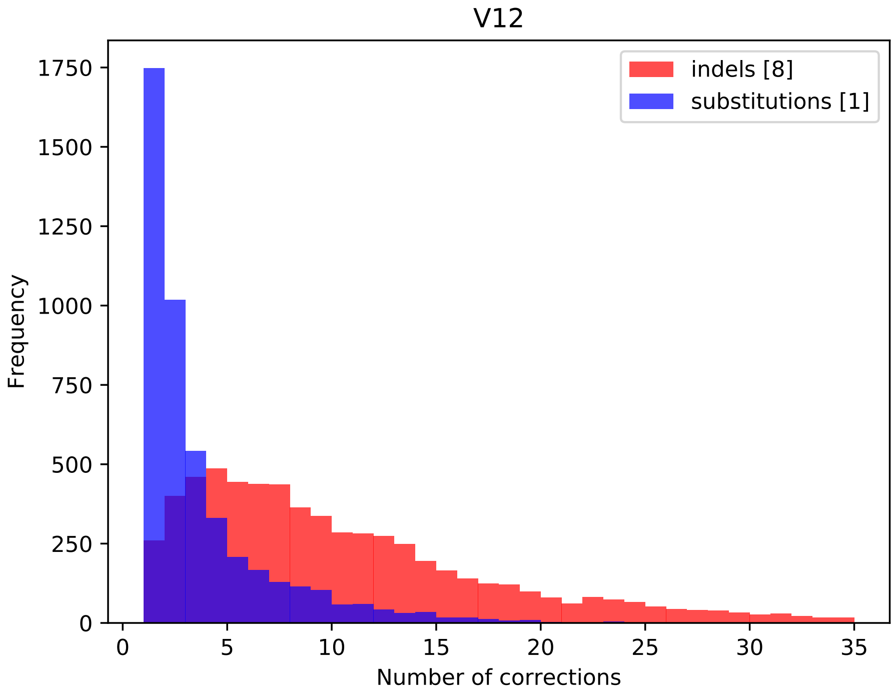

Figure C. Distribution of corrections in V12.

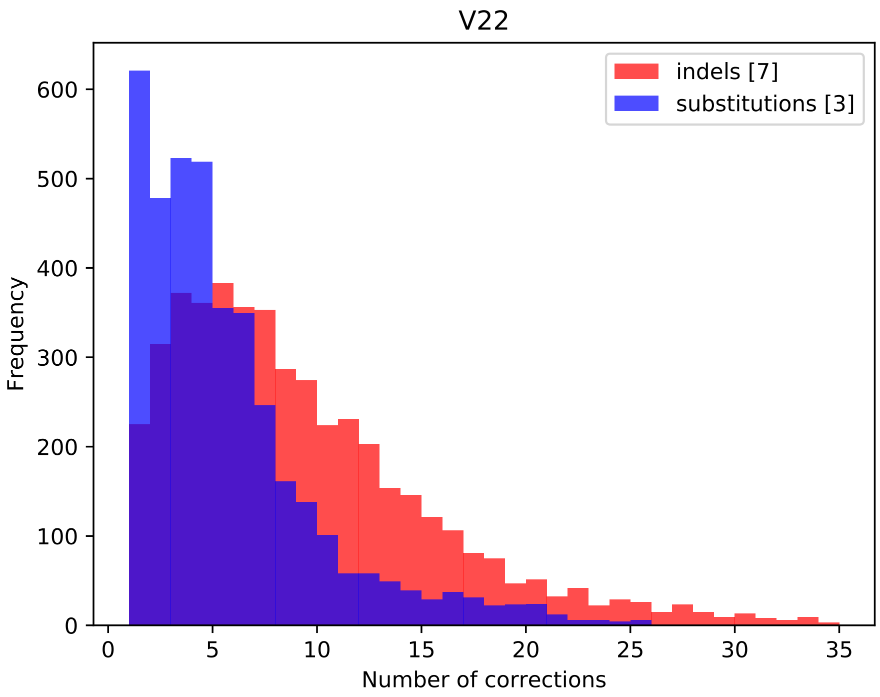

Figure D. Distribution of corrections in V22.

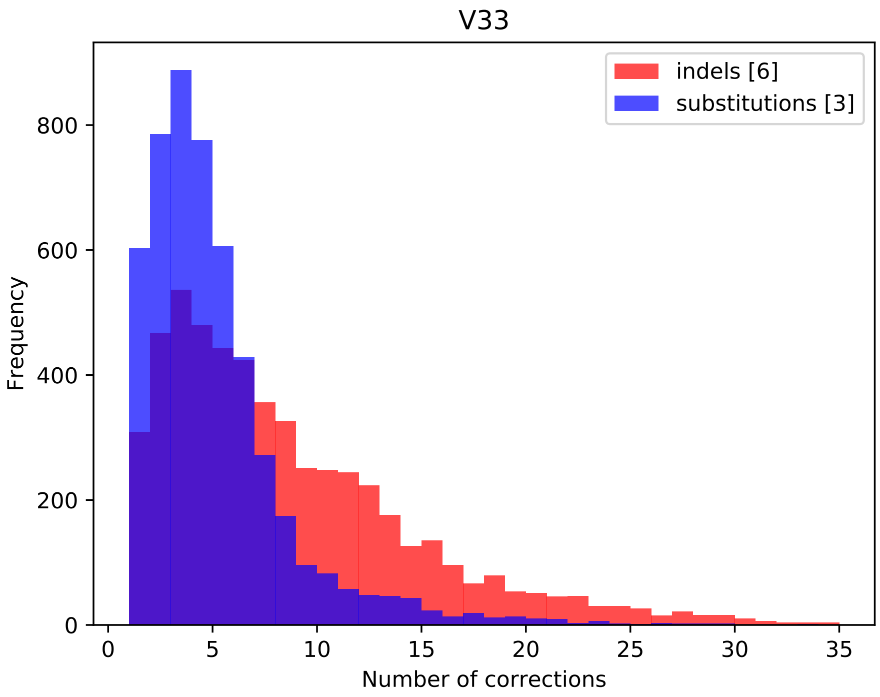

Figure E. Distribution of corrections in V33.

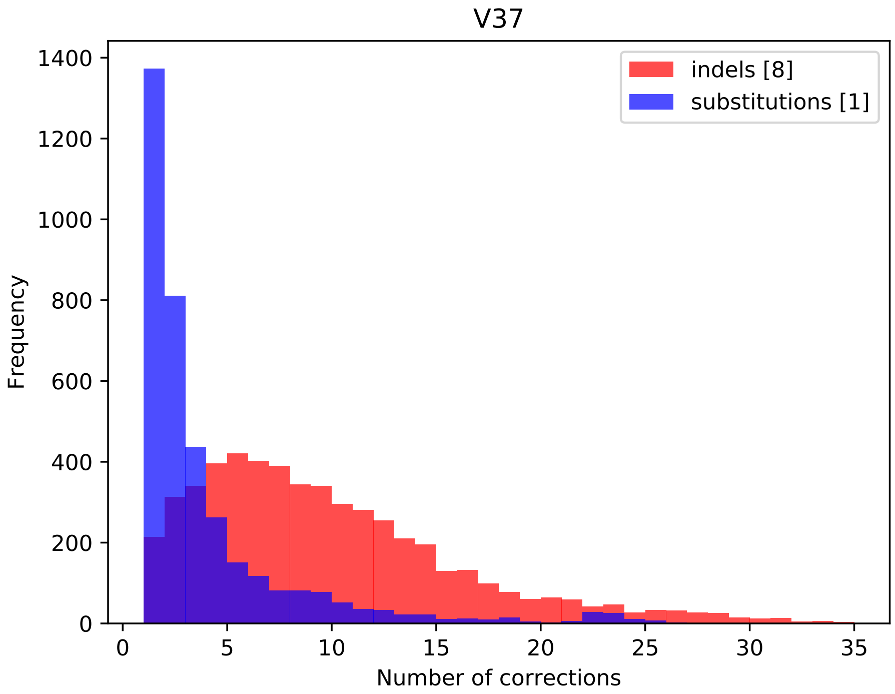

Figure F. Distribution of corrections in V37.

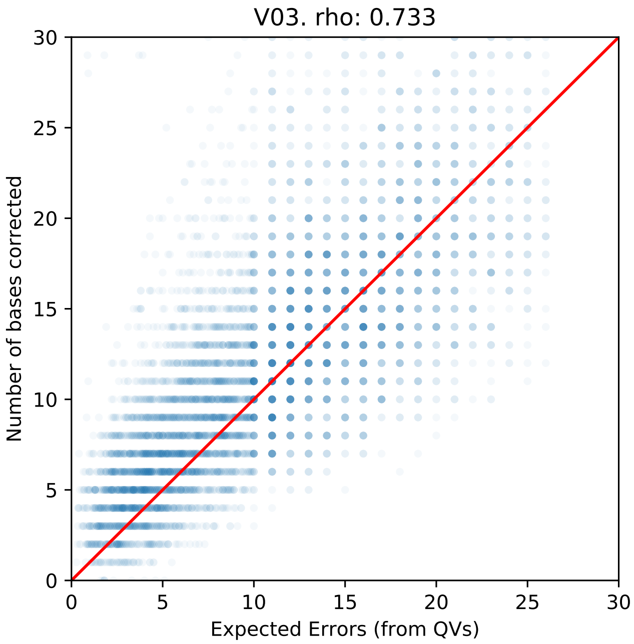

Figure G. Number of corrections versus expected number of errors in V03

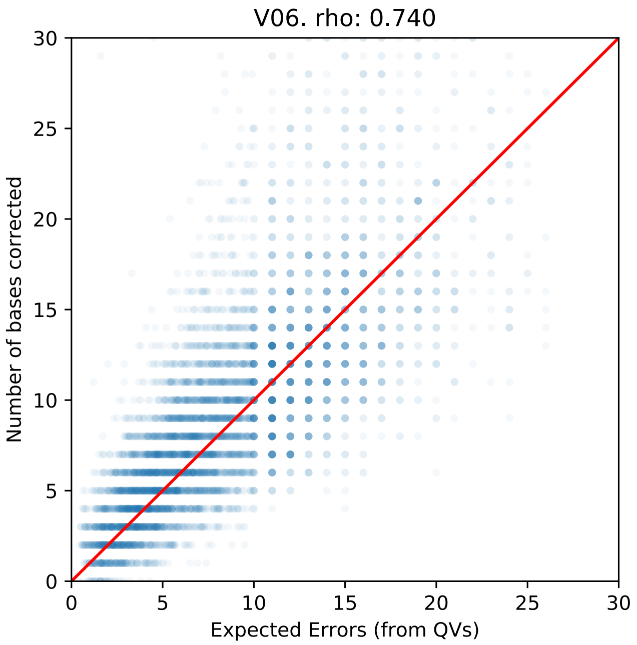

Figure H. Number of corrections versus expected number of errors in V06

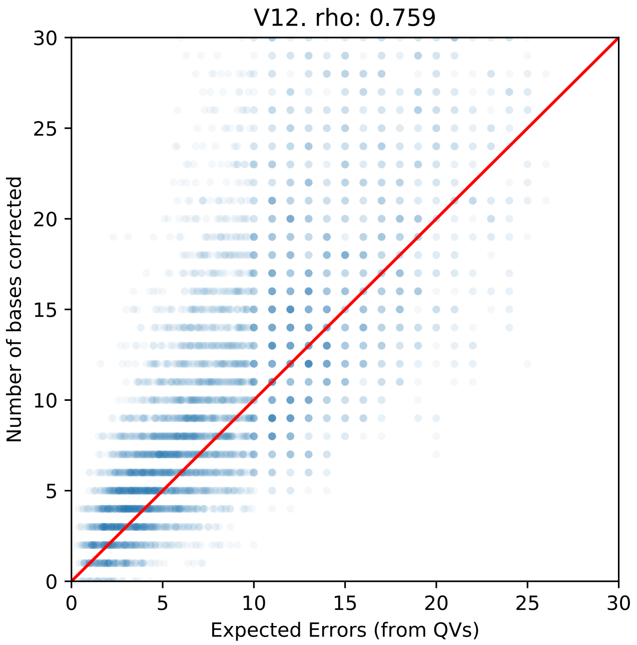

Figure I. Number of corrections versus expected number of errors in V12

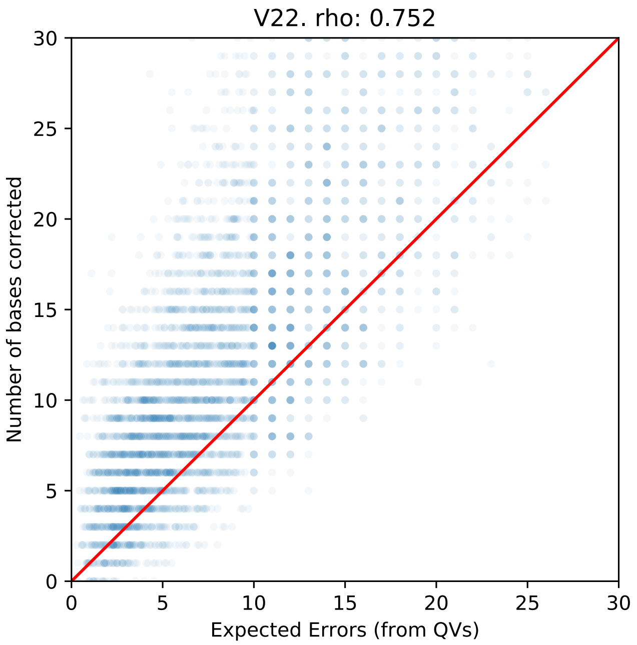

Figure J. Number of corrections versus expected number of errors in V22

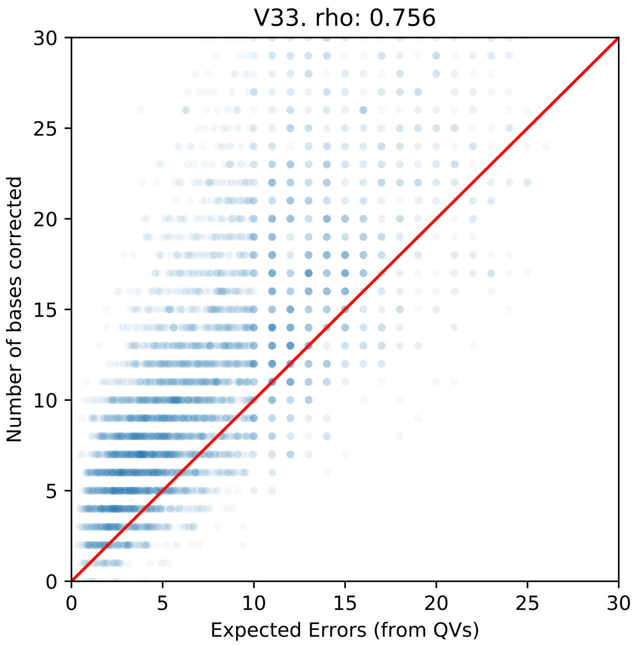

Figure K. Number of corrections versus expected number of errors in V33

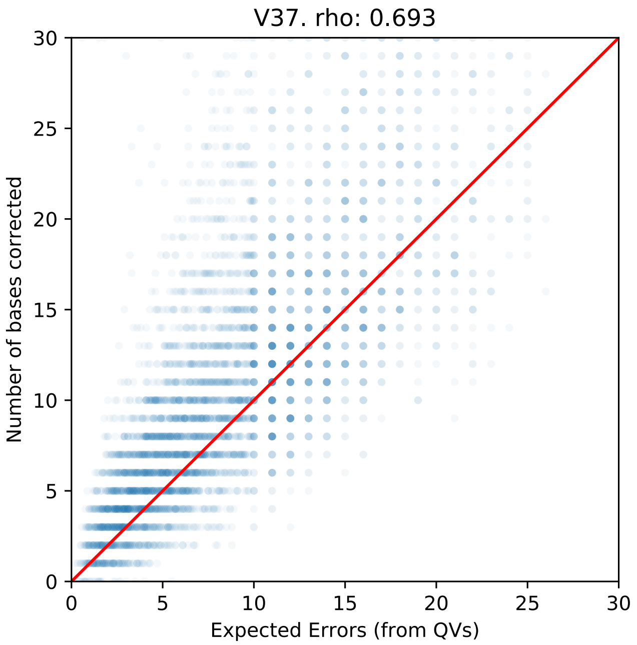

Figure L. Number of corrections versus expected number of errors in V37

Analysis of false negatives in simulation results

As shown in the main text, `FLEA` fails to recover some genuine sequences. All such failures are low-abundance sequences, where each makes up at most 1.6% of the population, and most make up much less. A breakdown appears in Table D. The fraction of the population represented by these false negatives at each time point is small: 10% of the population or less in most cases. Moreover, most of these templates differ from a `FLEA`-inferred sequence by only one base.

Fig. M plots abundance versus edit distance to nearest HQCS for all template sequence. The false negative sequences obviously have an edit distance ≥ 1, but the positives are also shown for reference. These plots confirm that the templates that `FLEA` fails to find tend to be extremely rare and also very similar to more high-abundance templates that were recovered.

Table D. False negatives by time point. "Total" is the number of true template sequences. False negatives are reported as n (x%), where n is the number of missing sequence, and x is their total abundance in the population. "Off-by-one" false negatives are sequences that do not appear in the `FLEA` results, but a sequence that differs by only one base does. "Remaining" false negatives differ by more than one base from any sequence in the `FLEA` results.

time point	total	false negatives	off-by-one	remaining
V03	127	65 (10.02%)	27 (6.57%)	38 (3.45%)
V06	147	52 (15.23%)	26 (7.77%)	26 (7.46%)
V12	104	16 (3.3%)	12 (2.55%)	4 (0.75%)
V22	137	26 (6.48%)	16 (4.38%)	10 (2.10%)
V33	80	35 (7.84%)	15 (4.67%)	20 (3.17%)
V37	79	19 (2.32%)	13 (1.61%)	6 (0.71%)

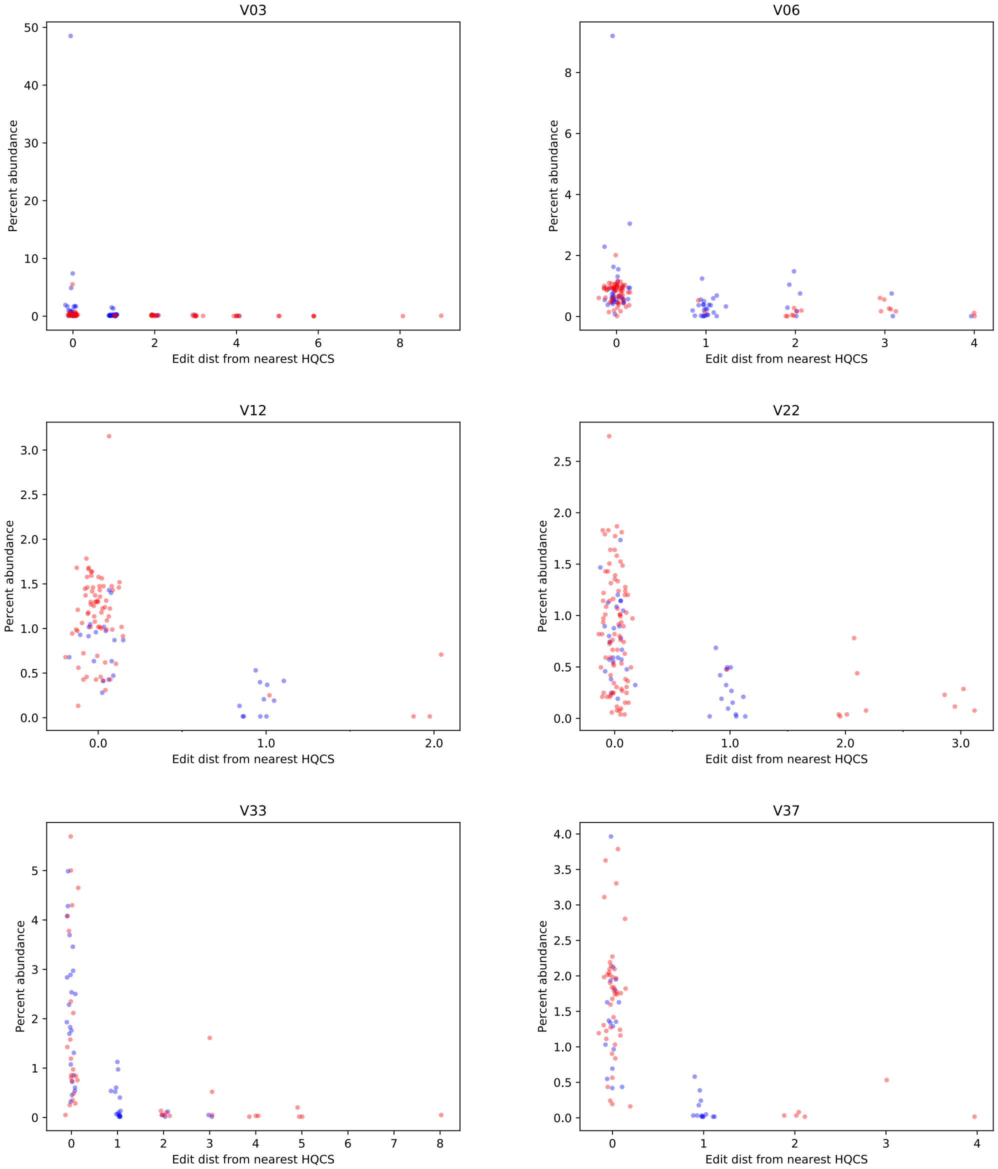

Figure M. From the N=10,000 simulation condition, for each time point, we depict the edit distance from each true template to the nearest HQCS. The Y axis displays the variant frequency (note: the number of reads is a Binomial variate from this, so many low abundance variants may generate no reads at all). In red, we color all true template sequences which are within 1bp of another true template.

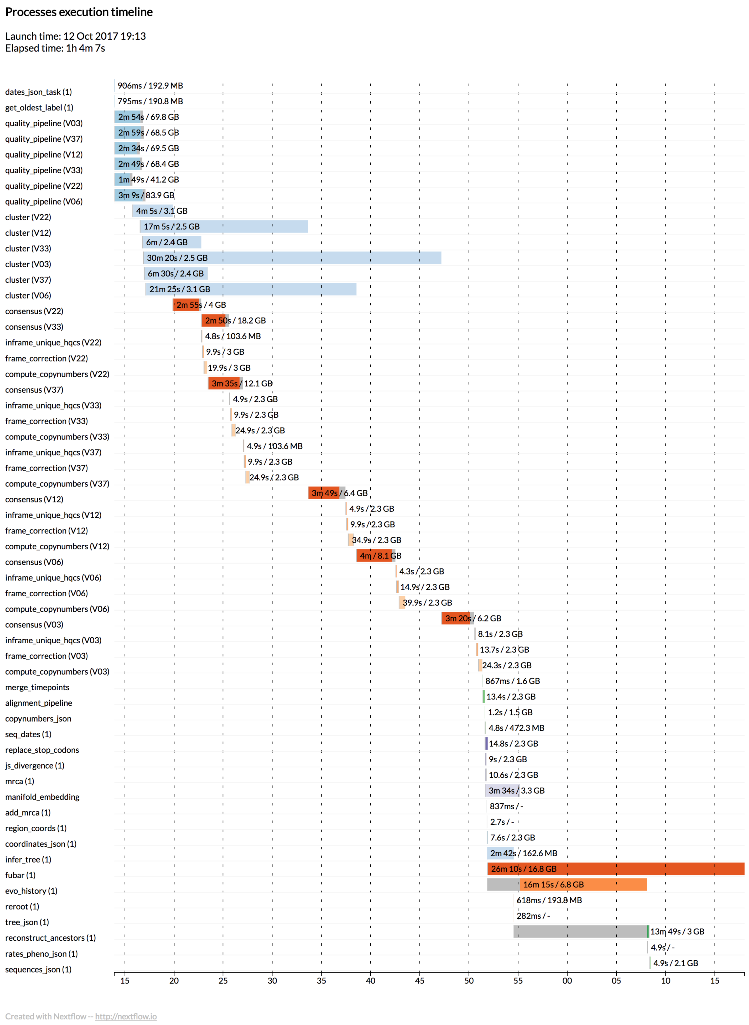

Figure N. Timeline of each task in the `FLEA` pipeline. Tasks are annotated with time per task and max memory used. Image generated with Nextflow's `-with-timeline` option.

Pipeline visualizations

Nextflow provides pipeline introspection and performance tools including tracing reports, task order graphs, and timeline visualizations (Fig. N).
